# Supplementary material for: Single-dose administration of therapeutic divalent siRNA targeting MECP2 prevents lethality for one year in an MECP2 duplication mouse model
Source: bioRxiv. 2025 Mar 29:2025.03.26.645328. Preprint. [Version 1] doi: 10.1101/2025.03.26.645328 (PMC11974818; doi:10.1101/2025.03.26.645328)
Supplement: Supplement 1 [file media-1.pdf]

Supplementary Materials and Methods
Oligonucleotide synthesis

Oligonucleotides were synthesized by phosphoramidite solid-phase synthesis on automated synthesizer using a MerMade12 (Biosearch Technologies, Novato, CA), Dr Oligo 48 (Biolytic, Fremont, CA) or AKTA Oligopilot 100 (Cytiva, Marlborough, MA). 5'-(E)-Vinyl tetraphosphonate (pivaloyloxymethyl) 2'-O-methyl-uridine 3'-CE phosphoramidite was used for the addition of 5’-Vinyl Phosphonate, 2′-F, 2′-OMe phosphoramidites with standard protecting groups were used to make the modified oligonucleotides. Phosphoramidites were dissolved at 0.1 M in anhydrous acetonitrile (ACN), with added anhydrous 15% dimethylformamide in the case of the 2'-OMe-Uridine amidite. 5-(Benzylthio)-1H-tetrazole (BTT) was used as the activator at 0.25 M. Coupling times were 4 minutes. Detritylations was performed using 3% trichloroacetic acid in dichloromethane or Toluene. Capping reagents used were CAP A (20% N-methylimidazole in ACN) and CAP B (20% acetic anhydride and 30% 2,6-lutidine in ACN). Phosphite oxidation to convert to phosphate or phosphorothioate was performed with 0.05 M iodine in pyridine-water (9:1, v/v) or 0.1 M solution of 3-[(dimethylaminomethylene)amino]-3H-1,2,4-dithiazole-5-thione (DDTT) in pyridine for 3 min, respectively. All reagents were purchased from Chemgenes, Wilmington, MA, phospohoramidites were purchased from Chemgenes and Hongene Biotech, Union City, CA. Oligonucleotides were grown on long-chain alkyl amine (LCAA) 500Å controlled pore glass (CPG) functionalized with Unylinker terminus for unconjugated oligonucleotides. Cholesterol conjugated oligonucleotides were synthesized on a 500Å LCAA-CPG support, tetraethyleneglycol cholesterol moiety was the functionalized group joined by a succinate linker to the support (Chemgenes). Divalent oligonucleotides were synthesized on a 1000Å LCAA-CPG support functionalized via succinyl linker with a glycerol-tetraethyleneglycol linker (Hongene Biotech).

Deprotection and purification of oligonucleotides for in vitro experiments

Oligonucleotides with or without cholesterol conjugate were cleaved and deprotected on-column with Ammonia gas (Airgas Specialty Gases). Briefly, columns were pre-wet with 100uL of water and immediately spun to remove the excess water. Columns were then placed in a reaction chamber (Biolytic) 90min at 65°C. A modified on-column ethanol precipitation protocol was used for desalting and counterion exchange. Briefly, 1mL of 0.1M sodium acetate in 80% ethanol is flushed through the column, followed by a rinse with 1mL 80% ethanol and finally after drying the excess ethanol, oligonucleotides were eluted with 600uL of water in 96 deep well plates.

Deprotection and Purification of Oligonucleotides for in vivo Experiments

5’-(E)-Vinyl-phosphonate containing oligonucleotides were cleaved and deprotected with 3% diethylamine in ammonium hydroxide, for 20 h at 35°C with agitation. Divalent oligonucleotides were cleaved and deprotected with 1:1 ammonium hydroxide and 40% aqueous monomethylamine, for 2 h at 25°C with slight agitation. The controlled pore glass was subsequently filtered and rinsed with 30 mL of 5% ACN in water and dried overnight by centrifugal vacuum concentration. Purifications were performed on an Agilent 1290 Infinity II HPLC system using Source 15Q anion exchange resin (Cytiva). The loading solution was 20 mM sodium acetate in 10% ACN in water, and elution solution was the loading solution with 1M

sodium bromide. Both oligonucleotide strands were eluted using a linear gradient from 30 to 70% in 40 min at 50°C. Peaks were monitored at 260nm. Pure fractions were combined and desalted by size exclusion using Sephadex G-25 resins (Cytiva). Oligonucleotides were then lyophilized and resuspended in water.

LC-MS analysis of oligonucleotides

Purity and identity of oligonucleotides were confirmed by IP-RP HPLC coupled to an Agilent 6530 Accurate-mass Q-TOF. LC parameters: buffer A: 100 mM 1,1,1,3,3,3-hexafluoroisopropanol (HFIP) (Oakwood Chemicals) and 9 mM triethylamine (TEA) (Fisher Scientific) in LC–MS grade water (Fisher Scientific); buffer B:100 mM HFIP and 9 mM TEA in LC–MS grade methanol (Fisher Scientific); column, Agilent AdvanceBio oligonucleotides C18; linear gradient 5–35% B 5min was used for unconjugated and divalent oligonucleotides; linear gradient 25–80% B 5min was used for cholesterol conjugated oligonucleotides; temperature, 60°C; flow rate, 0.85 ml/min. Peaks were monitored at 260nm. MS parameters: Source, electrospray ionization; ion polarity, negative mode; range, 100–3,200 m/z; scan rate, 2 spectra/s; capillary voltage, 4,000; fragmentor, 200 V; gas temp, 325°C.

ImageJ script for quantification of MECP2 in nuclei

```
//select directory to save output

 dir = getDirectory("Choose a Directory");
waitForUser("click on image you wish to analyze. make sure you have ROI set of nuclei already loaded. click ok to continue");

//calling ROI list of nuclei
n = roiManager('count');
for (i = 0; i < n; i++) {
roiManager('select', i);

//process roi here

run("Set Measurements...", "area mean modal min integrated median redirect=None decimal=3");
run("Measure");

//selectWindow("Summary");
}
selectWindow("Results");
Table.save(dir+File.separator+"results"+i+".csv"); //saves table
 waitForUser ("done, close Results table before next analysis.");
```

Animal Studies at JAX In Vivo Pharmacology Services

In-life Observations and Procedures

Complete Blinding of the study

Cage Cards, test article containers and Experimental Logs were blinded for treatment as “A” (Test article NTC), “B” (Test article M2), “C” (Test article M3), to blind the technicians performing injections, phenotypic scoring, body weight recording and behavioral tests for the dose injected. In addition, genotypes were also blinded.

Intracerebral Ventricular Injections (ICV) in Adult Mice

The mouse was anesthetized, and analgesia is administered.

The fur was removed, using clippers from the dorsal head in a roughly triangular area beginning ~ 3mm caudal to the nares and extending caudally to the cervical vertebrae. The lateral borders follow a line from the eye to the base of the pinna. Loose fur was removed with adhesive tape, dry gauze or gauze slightly dampened with ethanol. Ophthalmic ointment was placed on the eyes to prevent drying of the cornea. The skin was disinfected with a surgical scrub (iodine or chlorhexidine) and 70% ethanol using sterile swabs. Application of 70% ethanol starts in the center of the proposed incision site and works outward in ever widening circles to cover the entire clipped area. Using a new sterile swab, the surgical scrub was applied in the same manner. Repeat 70% ethanol and surgical scrub one additional time. The mouse was placed in ventral recumbency with the head secured on a stereotaxic device. A 1cm midsagittal skin incision was made on the scalp to expose the skull. The underlying fascia was separated, and the skin was retracted laterally. With a sterile cotton-tipped applicator soaked in 3% hydrogen peroxide, the skull was cleared of tissue down to the bone. The site was flushed with saline. The sterile drill was attached to the manipulator arm of the stereotaxic unit and the tip of the drill bit was aligned directly over bregma, a landmark on the skull and a stereotaxic reference point. Bregma was the position on the skull where the coronal and sagittal sutures intersect. The surgeon sets the X and Y coordinates of the stereotaxic device to zero. All subsequent stereotaxic coordinates reference these zero points. The manipulator arm with the attached drill is positioned at M/L +/-1.0, A/P -.40, and depth of 2.6 for injection (depth varies from 2.4 to 2.8). A hole was drilled through the skull but leaving the dura intact. The diameter of the drill bur was generally 0.6-1.6 mm. The site is flushed with sterile saline. The drill was removed and replaced on the manipulator arm with the sterile injector. The injector was lowered through the hole in the skull to the predetermined Z point. The siRNA was slowly injected, and the injector held in place for ~1 minute. Mice will receive a bilateral injection of 5 ul per ventricle for a total of 10 ul per mouse. 0.1% bupivacaine was applied to edges of the skin incision. The skin was closed with 6-0 suture with a swaged-on needle or tissue adhesive. Absorbable suture may be used to close skin incisions, but it was not preferred as it causes a greater inflammatory response than non-absorbable suture. Silk, plain gut, or chromic gut suture must never be used to close skin incisions. All wound closure material, except tissue adhesive or absorbable suture, must be removed after the wound has healed.

Open Field Test

Subjects were acclimated to the testing room for at least 60 min.

Equipment: Omnitech Versamax Open Field Arenas (40cmx 40cm x 40cm) with photobeams.

Mice were placed individually into the center of the arena. Data were recorded via a sensitive infrared (IR) photobeam three-dimensional grid system that is invisible to mice. When the mouse moves or travels, its body breaks the otherwise continuous beam. The automated system then translates the beam breaks into measurements such as distance traveled (cm), horizontal activity, vertical activity (rearing), and time spent in the center (anxiety-like) for 60 min.

Rotarod

Subjects were acclimated to the testing room for a minimum of 60 min.

Equipment: Ugo-Basile Accelerating Rotarod for Mice (model 47600).

Up to 5 mice were placed on the rod, which was rotating at the baseline speed of 5 RPM. Once all mice were placed on the rod, it accelerated linearly to 40 RPM over a period of 300 sec (5 min). Mice are run on three successive trials, and the latency to fall, which is measured as the time from the initiation of the 300 sec linear acceleration period to when the mouse falls off the rod into the tray below, is recorded for each mouse on each trial. Mice lasting 300 sec on the rod, without falling, receive a "latency to fall" time of 300 sec.

Nest building

Nesting behavior is an intrinsic behavior performed by male and female rodents that requires fine motor skills. The nest building test is a simple and versatile behavioral test suitable for the evaluation of motor deficits, cognitive decline as well as changes in general health or welfare. It is sensitive to environmental and physiological challenges, genetic mutations, and pharmacological interventions. There are several advantages of this test: it is performed in the home cage, without the presence of the experimenter, bedding material that is not used can be weighed to obtain an objective measure, and it is simple to perform while still yielding robust readouts. To test the individual nest building behavior, mice were individually housed in cages containing wood chip bedding and one square of pressed cotton `nestlet´. No other nesting material was provided. After 24hrs, photographs of the nests were taken, un-manipulated bedding material was weighed, and the manipulation of the nestlet and the quality of the built nest were analyzed manually according to a five-point scale.

Supplementary Figures

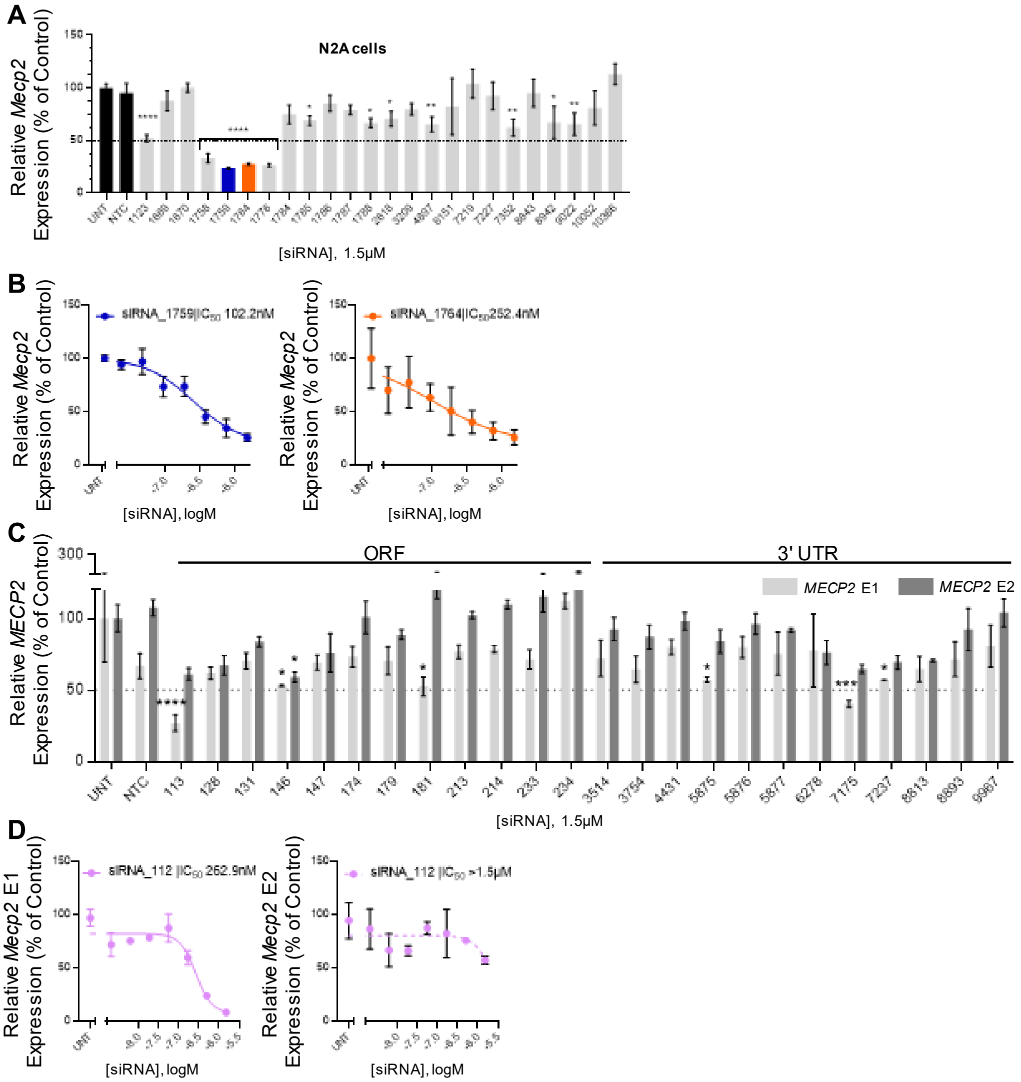

Supplementary Figure S1. **Lead siRNAs identified through in vitro screening**. (A) Relative *Mecp2* mRNA expression in mouse Neuro2A cells 72h post treatment with siRNAs at 1.5µM. (B) Relative *Mecp2* mRNA expression in mouse Neuro2A cells 72h post treatment with lead siRNAs in a seven-point dose response. (C) Relative *Mecp2* E1 (solid bars) and *Mecp2* E2 (patterned bars) mRNA expression in mouse Neuro2A cells 72h post treatment with siRNAs at 1.5µM. (D) Relative *Mecp2* E1 (left) and E2 (right) mRNA expression in mouse Neuro2A cells 72h post treatment with siRNA_112 in a seven-point dose response. Gene expression was measured using the QuantiGene™ Singleplex Assay Kit. Data represented as mean±s.e.m. of three independent replicates. Statistical analysis performed using Ordinary one-way ANOVA with Dunnett's adjustment for multiple comparisons.

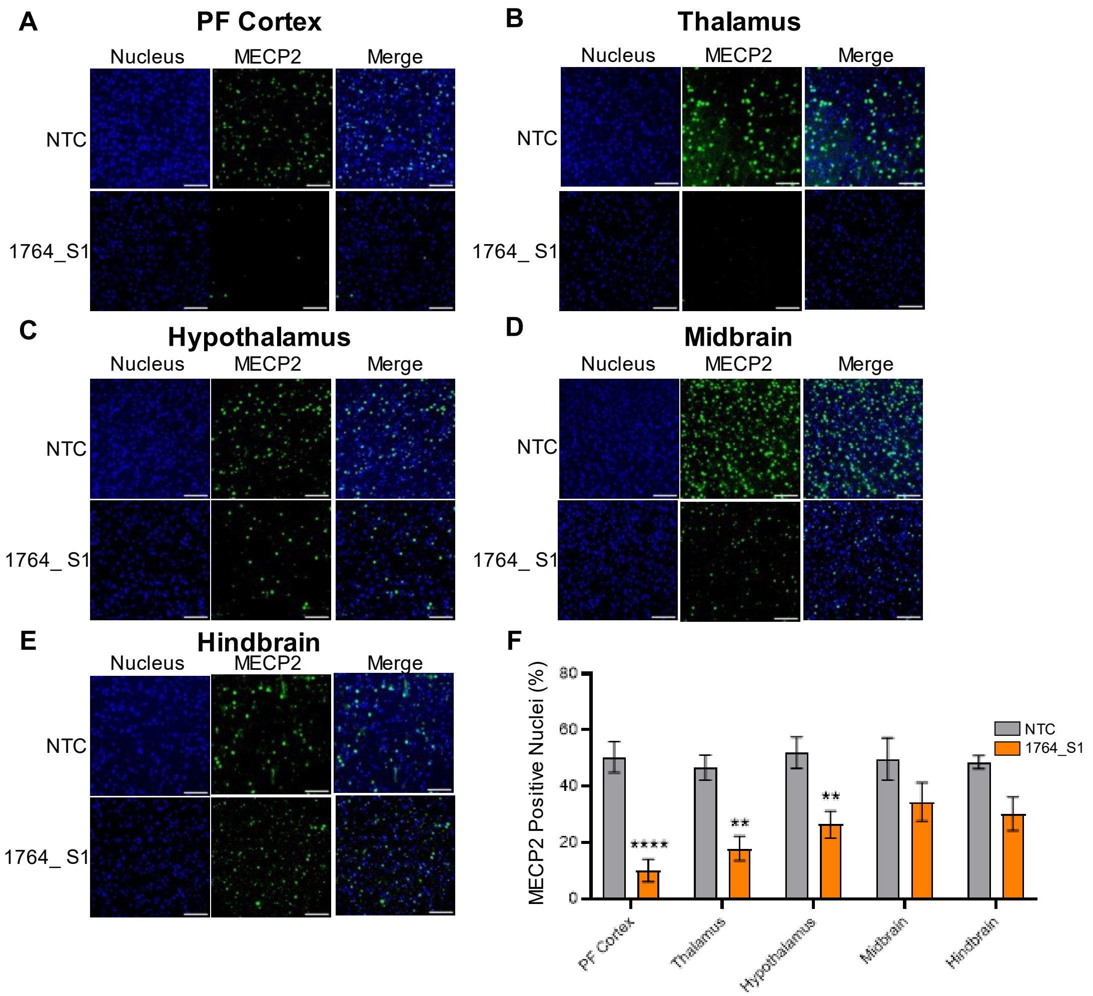

Supplementary Figure S2. Quantification of MECP2 positive nuclei in brain sections. Representative immunofluorescence images of DAPI and MECP2 stained sections of (A) prefrontal (PF) cortex, (B) thalamus, (C) hypothalamus, (D) midbrain, and (E) hindbrain along with (F) quantification of MECP2 positive nuclei. Data represented as mean±s.e.m. of individual animals. Immunofluorescence imaging was performed on sagittal brain sections stained with DAPI and anti-MECP2 antibody and imaged using Leica DMi8 widefield microscope. Quantification of MECP2 positive nuclei was performed using a custom imageJ script. Statistical analysis performed using two-way ANOVA with Dunnett's adjustment for multiple comparisons (**-p<0.01, ****-p<0.0001).

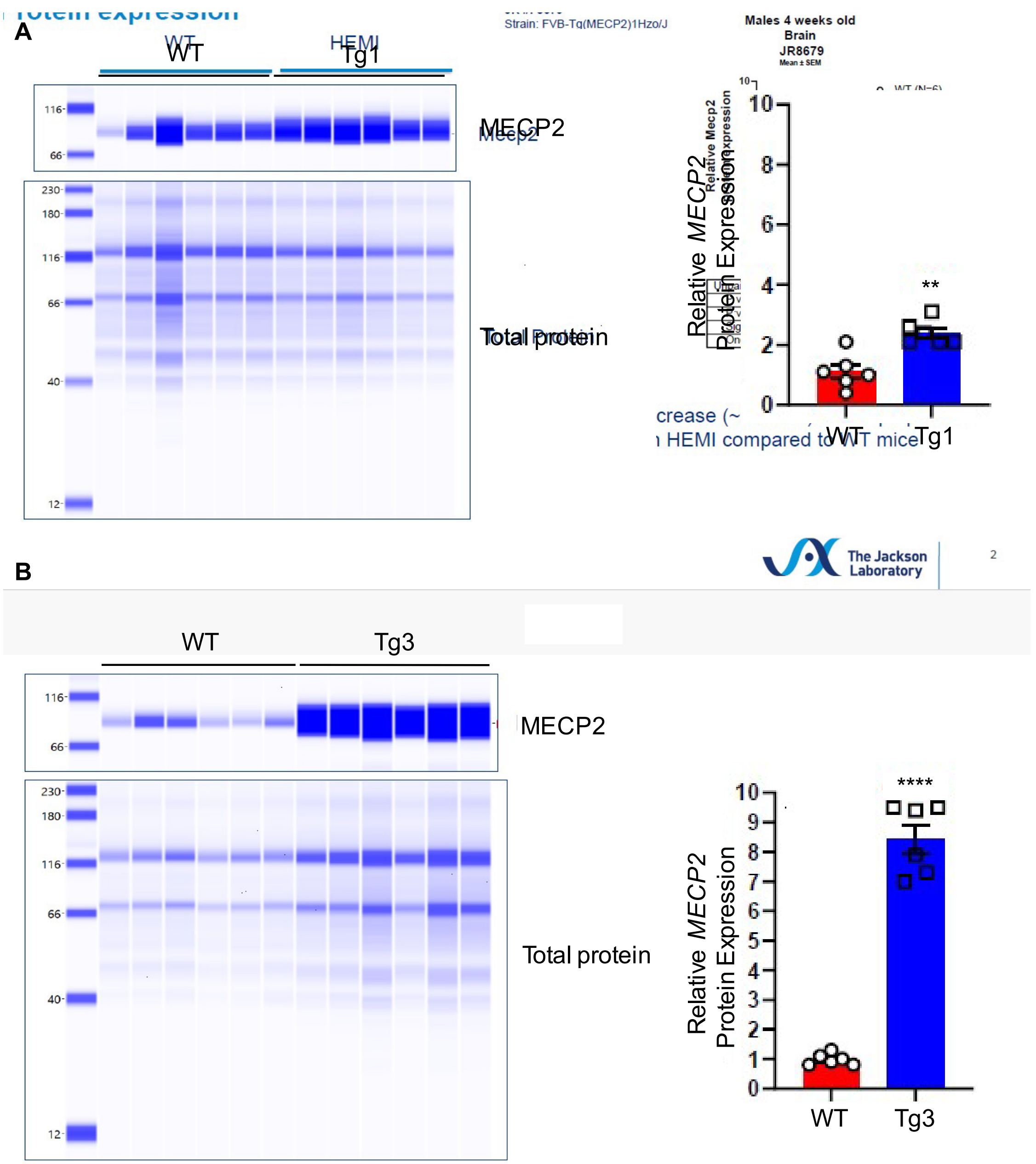

Supplementary Figure S3. Validation of Tg1 and Tg3 mouse models. Western blot gel images (left) and quantification of protein bands (right) from 4 weeks old (A) Tg1 mice and (B) Tg3 mice. Mecp2 protein bands were normalized to total protein. WT = FVB background, HEMI = transgenic strain. Data in bar graphs represented as mean±s.d. of individual animals (n=6). Statistical significance computed by unpaired t-test (**-p<0.01, ****-p<0.0001).

Supplementary Table S1. List of oligonucleotide sequences used in this study

Single strand ID	Oligo Name	Modified sequence
7051	MECP2_575_s	(mG)#(mA)#(mA)(mG)(fU)(fA)(fU)(mG)(fA)(mU)(mG)(mU)(mG)#(mU)#(mA)-TegChol
7087	MECP2_575_as	P(mU)#(fA)#(mC)(mA)(mC)(fA)(mU)(mC)(mA)(mU)(mA)(mC)(mU)#(fU)#(mC)#(fC)#(mC)#(mA)#(mG)#(fC)
7052	MECP2_572_s	(mU)#(mG)#(mG)(mG)(fA)(fA)(fG)(mU)(fA)(mU)(mG)(mA)(mU)#(mG)#(mA)-TegChol
7088	MECP2_572_as	P(mU)#(fC)#(mA)(mU)(mC)(fA)(mU)(mA)(mC)(mU)(mU)(mC)(mC)#(fC)#(mA)#(fG)#(mC)#(mA)#(mG)#(fA)
7053	MECP2_300_s	(mA)#(mA)#(mG)(mU)(fU)(fU)(fA)(mA)(fA)(mA)(mA)(mG)(mG)#(mU)#(mA)-TegChol
7089	MECP2_300_as	P(mU)#(fA)#(mC)(mC)(mU)(fU)(mU)(mU)(mU)(mA)(mA)(mA)(mC)#(fU)#(mU)#(fG)#(mA)#(mG)#(mG)#(fG)
7054	MECP2_827_s	(mC)#(mA)#(mC)(mG)(fU)(fC)(fA)(mG)(fA)(mG)(mG)(mG)(mU)#(mG)#(mA)-TegChol
7090	MECP2_827_as	P(mU)#(fC)#(mA)(mC)(mC)(fC)(mU)(mC)(mU)(mG)(mA)(mC)(mG)#(fU)#(mG)#(fG)#(mC)#(mC)#(mG)#(fC)
7055	MECP2_603_s	(mC)#(mA)#(mG)(mG)(fG)(fA)(fA)(mA)(fA)(mG)(mC)(mC)(mU)#(mU)#(mA)-TegChol
7091	MECP2_603_as	P(mU)#(fA)#(mA)(mG)(mG)(fC)(mU)(mU)(mU)(mU)(mC)(mC)(mC)#(fU)#(mG)#(fG)#(mG)#(mG)#(mA)#(fU)
7056	MECP2_887_s	(mC)#(mA)#(mA)(mG)(fA)(fU)(fG)(mC)(fC)(mU)(mU)(mU)(mU)#(mC)#(mA)-TegChol
7092	MECP2_887_as	P(mU)#(fG)#(mA)(mA)(mA)(fA)(mG)(mG)(mC)(mA)(mU)(mC)(mU)#(fU)#(mG)#(fA)#(mC)#(mA)#(mA)#(fG)
7057	MECP2_285_s	(mA)#(mA)#(mG)(mG)(fA)(fC)(fA)(mA)(fA)(mC)(mC)(mC)(mC)#(mU)#(mA)-TegChol
7093	MECP2_285_as	P(mU)#(fA)#(mG)(mG)(mG)(fG)(mU)(mU)(mU)(mG)(mU)(mC)(mC)#(fU)#(mU)#(fG)#(mA)#(mG)#(mG)#(fC)
7058	MECP2_605_s	(mG)#(mG)#(mG)(mA)(fA)(fA)(fA)(mG)(fC)(mC)(mU)(mU)(mU)#(mC)#(mA)-TegChol
7094	MECP2_605_as	P(mU)#(fG)#(mA)(mA)(mA)(fG)(mG)(mC)(mU)(mU)(mU)(mU)(mC)#(fC)#(mC)#(fU)#(mG)#(mG)#(mG)#(fG)
7059	MECP2_662_s	(mC)#(mA)#(mC)(mA)(fU)(fC)(fC)(mC)(fU)(mG)(mG)(mA)(mC)#(mC)#(mA)-TegChol

7095	MECP2_662_as	P(mU)#(fG)#(mG)(mU)(mC)(fC)(mA)(mG)(mG)(mG)(mA)(mU)(mG)#(fU)#(mG)#(fU)#(mC)#(mG)#(mC)#(fC)
7060	MECP2_535_s	(mC)#(mA)#(mC)(mG)(fG)(fA)(fA)(mG)(fC)(mU)(mU)(mA)(mA)#(mG)#(mA)-TegChol
7096	MECP2_535_as	P(mU)#(fC)#(mU)(mU)(mA)(fA)(mG)(mC)(mU)(mU)(mC)(mC)(mG)#(fU)#(mG)#(fU)#(mC)#(mC)#(mA)#(fG)
7061	MECP2_303_s	(mU)#(mU)#(mU)(mA)(fA)(fA)(fA)(mA)(fG)(mG)(mU)(mG)(mA)#(mA)#(mA)-TegChol
7097	MECP2_303_as	P(mU)#(fU)#(mU)(mC)(mA)(fC)(mC)(mU)(mU)(mU)(mU)(mU)(mA)#(fA)#(mA)#(fC)#(mU)#(mU)#(mG)#(fA)
7062	MECP2_895_s	(mC)#(mU)#(mU)(mU)(fU)(fC)(fA)(mA)(fA)(mC)(mU)(mU)(mC)#(mG)#(mA)-TegChol
7098	MECP2_895_as	P(mU)#(fC)#(mG)(mA)(mA)(fG)(mU)(mU)(mU)(mG)(mA)(mA)(mA)#(fA)#(mG)#(fG)#(mC)#(mA)#(mU)#(fC)
7063	MECP2_1776_s	(mA)#(mU)#(mU)(mA)(fA)(fC)(fU)(mG)(fA)(mA)(mA)(mU)(mA)#(mA)#(mA)-TegChol
7099	MECP2_1776_as	P(mU)#(fU)#(mU)(mA)(mU)(fU)(mU)(mC)(mA)(mG)(mU)(mU)(mA)#(fA)#(mU)#(fC)#(mG)#(mG)#(mG)#(fA)
7064	MECP2_1785_s	(mA)#(mA)#(mU)(mA)(fA)(fA)(fA)(mA)(fA)(mU)(mA)(mU)(mU)#(mU)#(mA)-TegChol
7100	MECP2_1785_as	P(mU)#(fA)#(mA)(mA)(mU)(fA)(mU)(mU)(mU)(mU)(mU)(mU)(mA)#(fU)#(mU)#(fU)#(mC)#(mA)#(mG)#(fU)
7065	MECP2_1759_s	(mU)#(mC)#(mU)(mG)(fA)(fC)(fA)(mA)(fA)(mG)(mC)(mU)(mU)#(mC)#(mA)-TegChol
7101	MECP2_1759_as	P(mU)#(fG)#(mA)(mA)(mG)(fC)(mU)(mU)(mU)(mG)(mU)(mC)(mA)#(fG)#(mA)#(fG)#(mC)#(mC)#(mC)#(fU)
7066	Mecp2_1123_s	(mC)#(mA)#(mA)(mA)(fA)(fA)(fG)(mA)(fA)(mA)(mG)(mC)(mC)#(mG)#(mA)-TegChol
7102	Mecp2_1123_as	P(mU)#(fC)#(mG)(mG)(mC)(fU)(mU)(mU)(mC)(mU)(mU)(mU)(mU)#(fU)#(mG)#(fG)#(mC)#(mC)#(mU)#(fC)
7067	MECP2_1787_s	(mU)#(mA)#(mA)(mA)(fA)(fA)(fA)(mU)(fA)(mU)(mU)(mU)(mU)#(mU)#(mA)-TegChol
7103	MECP2_1787_as	P(mU)#(fA)#(mA)(mA)(mA)(fA)(mU)(mA)(mU)(mU)(mU)(mU)(mU)#(fU)#(mA)#(fU)#(mU)#(mU)#(mC)#(fA)
7068	MECP2_1784_s	(mA)#(mA)#(mA)(mU)(fA)(fA)(fA)(mA)(fA)(mA)(mU)(mA)(mU)#(mU)#(mA)-TegChol
7104	MECP2_1784_as	P(mU)#(fA)#(mA)(mU)(mA)(fU)(mU)(mU)(mU)(mU)(mU)(mA)(mU)#(fU)#(mU)#(fC)#(mA)#(mG)#(mU)#(fU)

7069	MECP2_1786_s	(mA)#(mU)#(mA)(mA)(fA)(fA)(fA)(mA)(fU)(mA)(mU)(mU)(mU)#(mU)#(mA)-TegChol
7105	MECP2_1786_as	P(mU)#(fA)#(mA)(mA)(mA)(fU)(mA)(mU)(mU)(mU)(mU)(mU)(mU)#(fA)#(mU)#(fU)#(mU)#(mC)#(mA)#(fG)
7070	MECP2_1788_s	(mA)#(mA)#(mA)(mA)(fA)(fA)(fU)(mA)(fU)(mU)(mU)(mU)(mU)#(mU)#(mA)-TegChol
7106	MECP2_1788_as	P(mU)#(fA)#(mA)(mA)(mA)(fA)(mA)(mU)(mA)(mU)(mU)(mU)(mU)#(fU)#(mU)#(fA)#(mU)#(mU)#(mU)#(fC)
7071	MECP2_1758_s	(mC)#(mU)#(mC)(mU)(fG)(fA)(fC)(mA)(fA)(mA)(mG)(mC)(mU)#(mU)#(mA)-TegChol
7107	MECP2_1758_as	P(mU)#(fA)#(mA)(mG)(mC)(fU)(mU)(mU)(mG)(mU)(mC)(mA)(mG)#(fA)#(mG)#(fC)#(mC)#(mC)#(mU)#(fA)
7072	MECP2_1669_s	(mA)#(mU)#(mA)(mU)(fU)(fU)(fU)(mU)(fU)(mU)(mU)(mU)(mC)#(mU)#(mA)-TegChol
7108	MECP2_1669_as	P(mU)#(fA)#(mG)(mA)(mA)(fA)(mA)(mA)(mA)(mA)(mA)(mA)(mU)#(fA)#(mU)#(fU)#(mU)#(mU)#(mU)#(fU)
7073	MECP2_1670_s	(mU)#(mA)#(mU)(mU)(fU)(fU)(fU)(mU)(fU)(mU)(mU)(mC)(mU)#(mU)#(mA)-TegChol
7109	MECP2_1670_as	P(mU)#(fA)#(mA)(mG)(mA)(fA)(mA)(mA)(mA)(mA)(mA)(mA)(mA)#(fU)#(mA)#(fU)#(mU)#(mU)#(mU)#(fU)
7074	MECP2_1764_s	(mC)#(mA)#(mA)(mA)(fG)(fC)(fU)(mU)(fC)(mC)(mC)(mG)(mA)#(mU)#(mA)-TegChol
7110	MECP2_1764_as	P(mU)#(fA)#(mU)(mC)(mG)(fG)(mG)(mA)(mA)(mG)(mC)(mU)(mU)#(fU)#(mG)#(fU)#(mC)#(mA)#(mG)#(fA)
7075	Mecp2_4897_s	(mC)#(mA)#(mU)(mU)(fG)(fU)(fA)(mA)(fA)(mG)(mU)(mG)(mU)#(mG)#(mA)-TegChol
7111	Mecp2_4897_as	P(mU)#(fC)#(mA)(mC)(mA)(fC)(mU)(mU)(mU)(mA)(mC)(mA)(mA)#(fU)#(mG)#(fU)#(mU)#(mC)#(mA)#(fA)
7076	Mecp2_7219_s	(mU)#(mA)#(mU)(mA)(fU)(fA)(fU)(mA)(fU)(mA)(mU)(mC)(mU)#(mG)#(mA)-TegChol
7112	Mecp2_7219_as	P(mU)#(fC)#(mA)(mG)(mA)(fU)(mA)(mU)(mA)(mU)(mA)(mU)(mA)#(fU)#(mA)#(fU)#(mA)#(mU)#(mA)#(fU)
7077	Mecp2_10052_s	(mU)#(mU)#(mU)(mG)(fG)(fG)(fA)(mC)(fA)(mA)(mU)(mU)(mA)#(mC)#(mA)-TegChol
7113	Mecp2_10052_as	P(mU)#(fG)#(mU)(mA)(mA)(fU)(mU)(mG)(mU)(mC)(mC)(mC)(mA)#(fA)#(mA)#(fG)#(mC)#(mA)#(mC)#(fA)

7078	Mecp2_6151_s	(mA)#(mA)#(mA)(mA)(fA)(fU)(fU)(mA)(fU)(mA)(mU)(mU)(mU)#(mU)#(mA)-TegChol
7114	Mecp2_6151_as	P(mU)#(fA)#(mA)(mA)(mA)(fU)(mA)(mU)(mA)(mA)(mU)(mU)(mU)#(fU)#(mU)#(fU)#(mG)#(mU)#(mU)#(fA)
7079	Mecp2_3209_s	(mC)#(mC)#(mU)(mU)(fA)(fA)(fA)(mC)(fA)(mA)(mU)(mG)(mA)#(mG)#(mA)-TegChol
7115	Mecp2_3209_as	P(mU)#(fC)#(mU)(mC)(mA)(fU)(mU)(mG)(mU)(mU)(mU)(mA)(mA)#(fG)#(mG)#(fU)#(mC)#(mU)#(mG)#(fA)
7080	Mecp2_2618_s	(mA)#(mA)#(mG)(mG)(fA)(fA)(fA)(mA)(fU)(mA)(mU)(mU)(mC)#(mU)#(mA)-TegChol
7116	Mecp2_2618_as	P(mU)#(fA)#(mG)(mA)(mA)(fU)(mA)(mU)(mU)(mU)(mU)(mC)(mC)#(fU)#(mU)#(fU)#(mC)#(mU)#(mU)#(fC)
7081	Mecp2_9022_s	(mC)#(mA)#(mG)(mA)(fG)(fA)(fC)(mA)(fA)(mA)(mU)(mG)(mC)#(mU)#(mA)-TegChol
7117	Mecp2_9022_as	P(mU)#(fA)#(mG)(mC)(mA)(fU)(mU)(mU)(mG)(mU)(mC)(mU)(mC)#(fU)#(mG)#(fG)#(mA)#(mA)#(mC)#(fA)
7082	Mecp2_7352_s	(mA)#(mA)#(mG)(mG)(fG)(fA)(fA)(mA)(fA)(mA)(mA)(mU)(mU)#(mU)#(mA)-TegChol
7118	Mecp2_7352_as	P(mU)#(fA)#(mA)(mA)(mU)(fU)(mU)(mU)(mU)(mU)(mC)(mC)(mC)#(fU)#(mU)#(fG)#(mU)#(mC)#(mC)#(fU)
7083	Mecp2_10366_s	(mC)#(mA)#(mU)(mA)(fA)(fG)(fG)(mU)(fU)(mC)(mU)(mU)(mU)#(mU)#(mA)-TegChol
7119	Mecp2_10366_as	P(mU)#(fA)#(mA)(mA)(mA)(fG)(mA)(mA)(mC)(mC)(mU)(mU)(mA)#(fU)#(mG)#(fA)#(mA)#(mA)#(mA)#(fA)
7084	Mecp2_8843_s	(mU)#(mU)#(mG)(mG)(fU)(fU)(fU)(mU)(fA)(mU)(mU)(mU)(mU)#(mU)#(mA)-TegChol
7120	Mecp2_8843_as	P(mU)#(fA)#(mA)(mA)(mA)(fA)(mU)(mA)(mA)(mA)(mA)(mC)(mC)#(fA)#(mA)#(fA)#(mC)#(mC)#(mA)#(fA)
7085	Mecp2_8942_s	(mG)#(mU)#(mG)(mA)(fA)(fA)(fG)(mG)(fA)(mA)(mC)(mU)(mU)#(mU)#(mA)-TegChol
7121	Mecp2_8942_as	P(mU)#(fA)#(mA)(mA)(mG)(fU)(mU)(mC)(mC)(mU)(mU)(mU)(mC)#(fA)#(mC)#(fC)#(mC)#(mA)#(mC)#(fC)
7086	Mecp2_7227_s	(mU)#(mA)#(mU)(mC)(fU)(fG)(fU)(mA)(fU)(mA)(mU)(mU)(mU)#(mC)#(mA)-TegChol
7122	Mecp2_7227_as	P(mU)#(fG)#(mA)(mA)(mA)(fU)(mA)(mU)(mA)(mC)(mA)(mG)(mA)#(fU)#(mA)#(fU)#(mA)#(mU)#(mA)#(fU)
8174	MECP2_E1_s_111	(mA)#(mG)#(fG)(mA)(fG)(mG)(fA)(mG)(fA)(mG)(mA)(mC)(fU)#(mG)#(mA)-TegChol

8222	MECP2_E1_as_111	P(mU)#(fC)#(mA)(fG)(fU)(fC)(mU)(fC)(mU)(fC)(mC)(fU)(mC)#(fC)#(mU)#(fC)#(mG)#(mC)#(mC)#(fU)
8168	MECP2_E1_s_112	(mG)#(mG)#(fA)(mG)(fG)(mA)(fG)(mA)(fG)(mA)(mC)(mU)(fG)#(mG)#(mA)-TegChol
8216	MECP2_E1_as_112	P(mU)#(fC)#(mC)(fA)(fG)(fU)(mC)(fU)(mC)(fU)(mC)(fC)(mU)#(fC)#(mC)#(fU)#(mC)#(mG)#(mC)#(fC)
8169	MECP2_E1_s_113	(mG)#(mA)#(fG)(mG)(fA)(mG)(fA)(mG)(fA)(mC)(mU)(mG)(fG)#(mA)#(mA)-TegChol
8217	MECP2_E1_as_113	P(mU)#(fU)#(mC)(fC)(fA)(fG)(mU)(fC)(mU)(fC)(mU)(fC)(mC)#(fU)#(mC)#(fC)#(mU)#(mC)#(mG)#(fC)
8170	MECP2_E1_s_114	(mA)#(mG)#(fG)(mA)(fG)(mA)(fG)(mA)(fC)(mU)(mG)(mG)(fA)#(mA)#(mA)-TegChol
8218	MECP2_E1_as_114	P(mU)#(fU)#(mU)(fC)(fC)(fA)(mG)(fU)(mC)(fU)(mC)(fU)(mC)#(fC)#(mU)#(fC)#(mC)#(mU)#(mC)#(fG)
8173	MECP2_E1_s_115	(mG)#(mG)#(fA)(mG)(fA)(mG)(fA)(mC)(fU)(mG)(mG)(mA)(fA)#(mG)#(mA)-TegChol
8221	MECP2_E1_as_115	P(mU)#(fC)#(mU)(fU)(fC)(fC)(mA)(fG)(mU)(fC)(mU)(fC)(mU)#(fC)#(mC)#(fU)#(mC)#(mC)#(mU)#(fC)
8176	MECP2_E1_s_117	(mA)#(mG)#(fA)(mG)(fA)(mC)(fU)(mG)(fG)(mA)(mA)(mG)(fA)#(mA)#(mA)-TegChol
8224	MECP2_E1_as_117	P(mU)#(fU)#(mU)(fC)(fU)(fU)(mC)(fC)(mA)(fG)(mU)(fC)(mU)#(fC)#(mU)#(fC)#(mC)#(mU)#(mC)#(fC)
8175	MECP2_E1_s_118	(mG)#(mA)#(fG)(mA)(fC)(mU)(fG)(mG)(fA)(mA)(mG)(mA)(fA)#(mA)#(mA)-TegChol
8223	MECP2_E1_as_118	P(mU)#(fU)#(mU)(fU)(fC)(fU)(mU)(fC)(mC)(fA)(mG)(fU)(mC)#(fU)#(mC)#(fU)#(mC)#(mC)#(mU)#(fC)
8171	MECP2_E1_s_121	(mA)#(mC)#(fU)(mG)(fG)(mA)(fA)(mG)(fA)(mA)(mA)(mA)(fG)#(mU)#(mA)-TegChol
8219	MECP2_E1_as_121	P(mU)#(fA)#(mC)(fU)(fU)(fU)(mU)(fC)(mU)(fU)(mC)(fC)(mA)#(fG)#(mU)#(fC)#(mU)#(mC)#(mU)#(fC)
8166	MECP2_E1_s_122	(mC)#(mU)#(fG)(mG)(fA)(mA)(fG)(mA)(fA)(mA)(mA)(mG)(fU)#(mC)#(mA)-TegChol
8214	MECP2_E1_as_122	P(mU)#(fG)#(mA)(fC)(fU)(fU)(mU)(fU)(mC)(fU)(mU)(fC)(mC)#(fA)#(mG)#(fU)#(mC)#(mU)#(mC)#(fU)
8177	MECP2_E1_s_123	(mU)#(mG)#(fG)(mA)(fA)(mG)(fA)(mA)(fA)(mA)(mG)(mU)(fC)#(mA)#(mA)-TegChol
8225	MECP2_E1_as_123	P(mU)#(fU)#(mG)(fA)(fC)(fU)(mU)(fU)(mU)(fC)(mU)(fU)(mC)#(fC)#(mA)#(fG)#(mU)#(mC)#(mU)#(fC)

8164	MECP2_E1_s_126	(mA)#(mA)#(fG)(mA)(fA)(mA)(fA)(mG)(fU)(mC)(mA)(mG)(fA)#(mA)#(mA)-TegChol
8212	MECP2_E1_as_126	P(mU)#(fU)#(mU)(fC)(fU)(fG)(mA)(fC)(mU)(fU)(mU)(fU)(mC)#(fU)#(mU)#(fC)#(mC)#(mA)#(mG)#(fU)
8172	MECP2_E1_s_127	(mA)#(mG)#(fA)(mA)(fA)(mA)(fG)(mU)(fC)(mA)(mG)(mA)(fA)#(mG)#(mA)-TegChol
8220	MECP2_E1_as_127	P(mU)#(fC)#(mU)(fU)(fC)(fU)(mG)(fA)(mC)(fU)(mU)(fU)(mU)#(fC)#(mU)#(fU)#(mC)#(mC)#(mA)#(fG)
8155	MECP2_E1_s_1678	(mU)#(mU)#(fU)(mC)(fU)(mU)(fU)(mC)(fA)(mG)(mU)(mA)(fA)#(mA)#(mA)-TegChol
8203	MECP2_E1_as_1678	P(mU)#(fU)#(mU)(fU)(fA)(fC)(mU)(fG)(mA)(fA)(mA)(fG)(mA)#(fA)#(mA)#(fA)#(mA)#(mA)#(mA)#(fA)
8167	MECP2_E1_s_1679	(mU)#(mU)#(fC)(mU)(fU)(mU)(fC)(mA)(fG)(mU)(mA)(mA)(fA)#(mA)#(mA)-TegChol
8215	MECP2_E1_as_1679	P(mU)#(fU)#(mU)(fU)(fU)(fA)(mC)(fU)(mG)(fA)(mA)(fA)(mG)#(fA)#(mA)#(fA)#(mA)#(mA)#(mA)#(fA)
8159	MECP2_E1_s_1680	(mU)#(mC)#(fU)(mU)(fU)(mC)(fA)(mG)(fU)(mA)(mA)(mA)(fA)#(mA)#(mA)-TegChol
8207	MECP2_E1_as_1680	P(mU)#(fU)#(mU)(fU)(fU)(fU)(mA)(fC)(mU)(fG)(mA)(fA)(mA)#(fG)#(mA)#(fA)#(mA)#(mA)#(mA)#(fA)
8162	MECP2_E1_s_1681	(mC)#(mU)#(fU)(mU)(fC)(mA)(fG)(mU)(fA)(mA)(mA)(mA)(fA)#(mA)#(mA)-TegChol
8210	MECP2_E1_as_1681	P(mU)#(fU)#(mU)(fU)(fU)(fU)(mU)(fA)(mC)(fU)(mG)(fA)(mA)#(fA)#(mG)#(fA)#(mA)#(mA)#(mA)#(fA)
8158	MECP2_E1_s_1682	(mU)#(mU)#(fU)(mC)(fA)(mG)(fU)(mA)(fA)(mA)(mA)(mA)(fA)#(mA)#(mA)-TegChol
8206	MECP2_E1_as_1682	P(mU)#(fU)#(mU)(fU)(fU)(fU)(mU)(fU)(mA)(fC)(mU)(fG)(mA)#(fA)#(mA)#(fG)#(mA)#(mA)#(mA)#(fA)
8165	MECP2_E1_s_1683	(mU)#(mU)#(fC)(mA)(fG)(mU)(fA)(mA)(fA)(mA)(mA)(mA)(fA)#(mA)#(mA)-TegChol
8213	MECP2_E1_as_1683	P(mU)#(fU)#(mU)(fU)(fU)(fU)(mU)(fU)(mU)(fA)(mC)(fU)(mG)#(fA)#(mA)#(fA)#(mG)#(mA)#(mA)#(fA)
8163	MECP2_E1_s_1684	(mU)#(mC)#(fA)(mG)(fU)(mA)(fA)(mA)(fA)(mA)(mA)(mA)(fA)#(mA)#(mA)-TegChol
8211	MECP2_E1_as_1684	P(mU)#(fU)#(mU)(fU)(fU)(fU)(mU)(fU)(mU)(fU)(mA)(fC)(mU)#(fG)#(mA)#(fA)#(mA)#(mG)#(mA)#(fA)

8157	MECP2_E1_s_1685	(mC)#(mA)#(fG)(mU)(fA)(mA)(fA)(mA)(fA)(mA)(mA)(mA)(fA)#(mA)#(mA)-TegChol
8205	MECP2_E1_as_1685	P(mU)#(fU)#(mU)(fU)(fU)(fU)(mU)(fU)(mU)(fU)(mU)(fA)(mC)#(fU)#(mG)#(fA)#(mA)#(mA)#(mG)#(fA)
8160	MECP2_E1_s_1686	(mA)#(mG)#(fU)(mA)(fA)(mA)(fA)(mA)(fA)(mA)(mA)(mA)(fA)#(mA)#(mA)-TegChol
8208	MECP2_E1_as_1686	P(mU)#(fU)#(mU)(fU)(fU)(fU)(mU)(fU)(mU)(fU)(mU)(fU)(mA)#(fC)#(mU)#(fG)#(mA)#(mA)#(mA)#(fG)
8161	MECP2_E1_s_1687	(mG)#(mU)#(fA)(mA)(fA)(mA)(fA)(mA)(fA)(mA)(mA)(mA)(fA)#(mA)#(mA)-TegChol
8209	MECP2_E1_as_1687	P(mU)#(fU)#(mU)(fU)(fU)(fU)(mU)(fU)(mU)(fU)(mU)(fU)(mU)#(fA)#(mC)#(fU)#(mG)#(mA)#(mA)#(fA)
8156	MECP2_E1_s_1688	(mU)#(mA)#(fA)(mA)(fA)(mA)(fA)(mA)(fA)(mA)(mA)(mA)(fA)#(mA)#(mA)-TegChol
8204	MECP2_E1_as_1688	P(mU)#(fU)#(mU)(fU)(fU)(fU)(mU)(fU)(mU)(fU)(mU)(fU)(mU)#(fU)#(mA)#(fC)#(mU)#(mG)#(mA)#(fA)
8154	MECP2_E1_s_1689	(mA)#(mA)#(fA)(mA)(fA)(mA)(fA)(mA)(fA)(mA)(mA)(mA)(fA)#(mA)#(mA)-TegChol
8202	MECP2_E1_as_1689	P(mU)#(fU)#(mU)(fU)(fU)(fU)(mU)(fU)(mU)(fU)(mU)(fU)(mU)#(fU)#(mU)#(fA)#(mC)#(mU)#(mG)#(fA)
8180	MECP2_E2_s_113	(mG)#(mA)#(fG)(mG)(fA)(mG)(fA)(mG)(fA)(mC)(mU)(mG)(fC)#(mU)#(mA)-TegChol
8228	MECP2_E2_as_113	P(mU)#(fA)#(mG)(fC)(fA)(fG)(mU)(fC)(mU)(fC)(mU)(fC)(mC)#(fU)#(mC)#(fC)#(mU)#(mC)#(mG)#(fC)
8189	MECP2_E2_s_128	(mC)#(mA)#(fU)(mA)(fA)(mA)(fA)(mA)(fU)(mA)(mC)(mA)(fG)#(mA)#(mA)-TegChol
8237	MECP2_E2_as_128	P(mU)#(fU)#(mC)(fU)(fG)(fU)(mA)(fU)(mU)(fU)(mU)(fU)(mA)#(fU)#(mG)#(fG)#(mA)#(mG)#(mC)#(fA)
8182	MECP2_E2_s_131	(mA)#(mA)#(fA)(mA)(fA)(mU)(fA)(mC)(fA)(mG)(mA)(mC)(fU)#(mC)#(mA)-TegChol
8230	MECP2_E2_as_131	P(mU)#(fG)#(mA)(fG)(fU)(fC)(mU)(fG)(mU)(fA)(mU)(fU)(mU)#(fU)#(mU)#(fA)#(mU)#(mG)#(mG)#(fA)
8181	MECP2_E2_s_146	(mC)#(mC)#(fA)(mG)(fU)(mU)(fC)(mC)(fU)(mG)(mC)(mU)(fU)#(mU)#(mA)-TegChol
8229	MECP2_E2_as_146	P(mU)#(fA)#(mA)(fA)(fG)(fC)(mA)(fG)(mG)(fA)(mA)(fC)(mU)#(fG)#(mG)#(fU)#(mG)#(mA)#(mG)#(fU)
8188	MECP2_E2_s_147	(mC)#(mA)#(fG)(mU)(fU)(mC)(fC)(mU)(fG)(mC)(mU)(mU)(fU)#(mG)#(mA)-TegChol

8236	MECP2_E2_as_147	P(mU)#(fC)#(mA)(fA)(fA)(fG)(mC)(fA)(mG)(fG)(mA)(fA)(mC)#(fU)#(mG)#(fG)#(mU)#(mG)#(mA)#(fG)
8184	MECP2_E2_s_174	(mC)#(mU)#(fC)(mC)(fC)(mC)(fA)(mG)(fA)(mA)(mU)(mA)(fC)#(mA)#(mA)-TegChol
8232	MECP2_E2_as_174	P(mU)#(fU)#(mG)(fU)(fA)(fU)(mU)(fC)(mU)(fG)(mG)(fG)(mG)#(fA)#(mG)#(fU)#(mC)#(mA)#(mC)#(fA)
8179	MECP2_E2_s_179	(mC)#(mA)#(fG)(mA)(fA)(mU)(fA)(mC)(fA)(mC)(mC)(mU)(fU)#(mG)#(mA)-TegChol
8227	MECP2_E2_as_179	P(mU)#(fC)#(mA)(fA)(fG)(fG)(mU)(fG)(mU)(fA)(mU)(fU)(mC)#(fU)#(mG)#(fG)#(mG)#(mG)#(mA)#(fG)
8178	MECP2_E2_s_181	(mG)#(mA)#(fA)(mU)(fA)(mC)(fA)(mC)(fC)(mU)(mU)(mG)(fC)#(mU)#(mA)-TegChol
8226	MECP2_E2_as_181	P(mU)#(fA)#(mG)(fC)(fA)(fA)(mG)(fG)(mU)(fG)(mU)(fA)(mU)#(fU)#(mC)#(fU)#(mG)#(mG)#(mG)#(fG)
8183	MECP2_E2_s_213	(mC)#(mA)#(fG)(mG)(fA)(mU)(fU)(mC)(fC)(mA)(mU)(mG)(fG)#(mU)#(mA)-TegChol
8231	MECP2_E2_as_213	P(mU)#(fA)#(mC)(fC)(fA)(fU)(mG)(fG)(mA)(fA)(mU)(fC)(mC)#(fU)#(mG)#(fU)#(mU)#(mG)#(mG)#(fA)
8186	MECP2_E2_s_214	(mA)#(mG)#(fG)(mA)(fU)(mU)(fC)(mC)(fA)(mU)(mG)(mG)(fU)#(mA)#(mA)-TegChol
8234	MECP2_E2_as_214	P(mU)#(fU)#(mA)(fC)(fC)(fA)(mU)(fG)(mG)(fA)(mA)(fU)(mC)#(fC)#(mU)#(fG)#(mU)#(mU)#(mG)#(fG)
8185	MECP2_E2_s_233	(mG)#(mA)#(fU)(mG)(fU)(mU)(fA)(mG)(fG)(mG)(mC)(mU)(fC)#(mA)#(mA)-TegChol
8233	MECP2_E2_as_233	P(mU)#(fU)#(mG)(fA)(fG)(fC)(mC)(fC)(mU)(fA)(mA)(fC)(mA)#(fU)#(mC)#(fC)#(mC)#(mA)#(mG)#(fC)
8187	MECP2_E2_s_234	(mA)#(mU)#(fG)(mU)(fU)(mA)(fG)(mG)(fG)(mC)(mU)(mC)(fA)#(mG)#(mA)-TegChol
8235	MECP2_E2_as_234	P(mU)#(fC)#(mU)(fG)(fA)(fG)(mC)(fC)(mC)(fU)(mA)(fA)(mC)#(fA)#(mU)#(fC)#(mC)#(mC)#(mA)#(fG)
8201	MECP2_E2_s_3514	(mA)#(mA)#(fU)(mG)(fG)(mC)(fA)(mA)(fU)(mG)(mU)(mU)(fU)#(mU)#(mA)-TegChol
8249	MECP2_E2_as_3514	P(mU)#(fA)#(mA)(fA)(fA)(fC)(mA)(fU)(mU)(fG)(mC)(fC)(mA)#(fU)#(mU)#(fC)#(mA)#(mA)#(mG)#(fA)
8197	MECP2_E2_s_3754	(mU)#(mA)#(fU)(mA)(fU)(mC)(fU)(mA)(fA)(mA)(mU)(mC)(fU)#(mG)#(mA)-TegChol
8245	MECP2_E2_as_3754	P(mU)#(fC)#(mA)(fG)(fA)(fU)(mU)(fU)(mA)(fG)(mA)(fU)(mA)#(fU)#(mA)#(fA)#(mG)#(mA)#(mG)#(fA)

8193	MECP2_E2_s_4431	(mU)#(mU)#(fU)(mU)(fU)(mA)(fU)(mG)(fU)(mA)(mU)(mU)(fA)#(mU)#(mA)-TegChol
8241	MECP2_E2_as_4431	P(mU)#(fA)#(mU)(fA)(fA)(fU)(mA)(fC)(mA)(fU)(mA)(fA)(mA)#(fA)#(mA)#(fC)#(mC)#(mC)#(mA)#(fA)
8200	MECP2_E2_s_5875	(mG)#(mC)#(fA)(mU)(fA)(mU)(fA)(mC)(fA)(mU)(mU)(mU)(fU)#(mU)#(mA)-TegChol
8248	MECP2_E2_as_5875	P(mU)#(fA)#(mA)(fA)(fA)(fA)(mU)(fG)(mU)(fA)(mU)(fA)(mU)#(fG)#(mC)#(fC)#(mC)#(mA)#(mA)#(fA)
8190	MECP2_E2_s_5876	(mC)#(mA)#(fU)(mA)(fU)(mA)(fC)(mA)(fU)(mU)(mU)(mU)(fU)#(mA)#(mA)-TegChol
8238	MECP2_E2_as_5876	P(mU)#(fU)#(mA)(fA)(fA)(fA)(mA)(fU)(mG)(fU)(mA)(fU)(mA)#(fU)#(mG)#(fC)#(mC)#(mC)#(mA)#(fA)
8198	MECP2_E2_s_5877	(mA)#(mU)#(fA)(mU)(fA)(mC)(fA)(mU)(fU)(mU)(mU)(mU)(fA)#(mG)#(mA)-TegChol
8246	MECP2_E2_as_5877	P(mU)#(fC)#(mU)(fA)(fA)(fA)(mA)(fA)(mU)(fG)(mU)(fA)(mU)#(fA)#(mU)#(fG)#(mC)#(mC)#(mC)#(fA)
8199	MECP2_E2_s_6278	(mC)#(mA)#(fG)(mA)(fA)(mA)(fA)(mU)(fU)(mA)(mC)(mA)(fU)#(mU)#(mA)-TegChol
8247	MECP2_E2_as_6278	P(mU)#(fA)#(mA)(fU)(fG)(fU)(mA)(fA)(mU)(fU)(mU)(fU)(mC)#(fU)#(mG)#(fC)#(mC)#(mA)#(mA)#(fA)
8196	MECP2_E2_s_7175	(mU)#(mA)#(fU)(mU)(fG)(mC)(fA)(mC)(fA)(mA)(mU)(mU)(fA)#(mU)#(mA)-TegChol
8244	MECP2_E2_as_7175	P(mU)#(fA)#(mU)(fA)(fA)(fU)(mU)(fG)(mU)(fG)(mC)(fA)(mA)#(fU)#(mA)#(fU)#(mA)#(mC)#(mA)#(fG)
8195	MECP2_E2_s_7237	(mA)#(mA)#(fU)(mU)(fA)(mU)(fA)(mC)(fC)(mU)(mG)(mU)(fU)#(mG)#(mA)-TegChol
8243	MECP2_E2_as_7237	P(mU)#(fC)#(mA)(fA)(fC)(fA)(mG)(fG)(mU)(fA)(mU)(fA)(mA)#(fU)#(mU)#(fU)#(mU)#(mA)#(mA)#(fC)
8191	MECP2_E2_s_8813	(mG)#(mU)#(fG)(mA)(fA)(mA)(fG)(mG)(fA)(mA)(mU)(mU)(fU)#(mU)#(mA)-TegChol
8239	MECP2_E2_as_8813	P(mU)#(fA)#(mA)(fA)(fA)(fU)(mU)(fC)(mC)(fU)(mU)(fU)(mC)#(fA)#(mC)#(fC)#(mC)#(mA)#(mC)#(fC)
8194	MECP2_E2_s_8893	(mC)#(mA)#(fG)(mA)(fG)(mA)(fC)(mA)(fA)(mA)(mU)(mA)(fU)#(mU)#(mA)-TegChol
8242	MECP2_E2_as_8893	P(mU)#(fA)#(mA)(fU)(fA)(fU)(mU)(fU)(mG)(fU)(mC)(fU)(mC)#(fU)#(mG)#(fG)#(mA)#(mA)#(mC)#(fA)

8192	MECP2_E2_s_9967	(mU)#(mU)#(fU)(mG)(fG)(mG)(fA)(mC)(fA)(mA)(mU)(mU)(fA)#(mC)#(mA)-TegChol
8240	MECP2_E2_as_9967	P(mU)#(fG)#(mU)(fA)(fA)(fU)(mU)(fG)(mU)(fC)(mC)(fC)(mA)#(fA)#(mA)#(fA)#(mC)#(mA)#(mC)#(fA)

P=phosphate, #=phosphorothioate, m=2'-Omethyl, f=2'-fluoro, A=adenine, U=uracil, G=guanine, C=cytosine, TegChol = cholesterol conjugate with linker
